# Moving beyond projects: a logic model evaluation of an established translational simulation program

**DOI:** 10.1186/s41077-026-00434-x

**Published:** 2026-04-02

**Authors:** Andrew Petrosoniak, Aradhana Tewari, Lindsay Beavers, Nivetha Chandran, Kristen Sampson, Christine Léger, Nazanin Khodadoust, Ryan Brydges

**Affiliations:** 1 Allan Waters Family Simulation Centre, St. Michael’s Hospital, Unity Health Toronto, Toronto, Canada; 2 Li Ka Shing Knowledge Institute, 209 Victoria St., Toronto, ON Canada; 3 Division of Emergency Medicine, Department of Medicine, University of Toronto, Toronto, ON Canada; 4 Department of Physical Therapy, Temerty Faculty of Medicine, University of Toronto, Toronto, ON Canada; 5 Technology-Enabled Education, Education Portfolio, Unity Health Toronto, Toronto, ON Canada; 6 Department of Medicine, University of Toronto, Toronto, ON Canada

**Keywords:** Translational Simulation, System change, Logic Model, Program evaluation, Healthcare Simulation

## Abstract

**Background:**

Advancing beyond a sole focus on individuals’ and team’s clinical skills training, translational simulation uses simulation-based methodologies to diagnose healthcare system gaps and inform targeted quality and safety improvements. While promising conceptually, few reports exist on how to build sustainable, system-level translational simulation programs in hospital settings.

**Methods:**

We applied a logic model evaluation framework to assess four-years of activity in Unity Health Toronto’s established translational simulation program. Researchers not directly involved in the translational simulation program interviewed internal team members and program partners who had utilized the program. The interviews focused on the processes associated with project selection, implementation, and outcome collection. The research team used an iterative, team-based consensus approach to develop a fulsome logic model to richly describe the observed impacts and challenges of the program.

**Results:**

Our analysis reflected experiences of 10 internal team members, and 14 program partners who contributed to 11 completed projects. We found that early collaboration during project intake was critical, but sometimes slowed perceived progress. Program members described a regular need to establish a shared conceptual understanding and clear roles at the outset with each program partner. Mutual adaptability between the simulation team and partners was reported as a defining factor for successful project implementation. Program outputs primarily consisted of simulation team generated reports, which generally met partner expectations; however, several partners desired more detailed, actionable analyses. Mid- to long-term outcomes varied based on project goals, including changes to clinical workflows, policy revisions, improved team performance, and enhanced patient outcomes.

**Conclusions:**

Grounded in refined claims for each logic model element, this evaluation provides transferable principles and data-driven recommendations for implementing and sustaining translational simulation programs. Our findings informed local program refinements while also producing strategies that can be applied broadly to translational simulation programs: 1) Flexible engagement tailored to partner needs and context 2) Clear definitions of simulation modalities, milestones, roles, objectives, and deliverables, 3) Structured communication throughout all project phases, and 4) Consistent follow-up to ensure translation of insights into clear returns on investment, and sustained system change.

**Supplementary Information:**

The online version contains supplementary material available at 10.1186/s41077-026-00434-x.

Healthcare simulation has become entrenched as a modality for training and education across cognitive, procedural, communication, and teamwork domains [1]. Over the past decade, simulation has evolved beyond its pedagogical roots to encompass a broader systems-oriented function [2]. Referred to as *translational simulation*, educators and system leaders have been deliberately using simulation to improve healthcare quality and safety by diagnosing system issues and delivering interventional strategies that target the patient, system, and organizational-levels [3]. 

The growing enthusiasm for using translational simulation to improve healthcare systems has been met with growing pains in how to agree on core concepts and operationalize them into logistics that impact healthcare organizations at scale [4]. As discussed previously for all simulation modalities [5, 6], we align with the originators of translational simulation [2, 4] who emphasize focusing on its function—to deliver diagnostic and interventional insights that directly enhance healthcare quality and patient safety—rather than on its location, modality, or technology. Unfortunately, some inconsistent and varied applications of translational simulation (e.g., use of multiple and confusing terms, like ‘transformative simulation’ or “in-situ simulation”) have challenged simulation leaders’ and healthcare organizations’ efforts to systematically embed and operationalize translational simulation within their regular practices.

Searches of ‘translational simulation’ in the existing literature will generate several conceptual frameworks [3, 4] and case studies [7, 8] illustrating its potential value; however, most studies remain descriptive, narrowly focused, and/or difficult to replicate across healthcare systems. Fortunately, researchers have been gradually providing examples of translational simulation that result in tangible and meaningful, patient-oriented outcomes [9]. For example, programs have used translational simulation to reduce time to blood for trauma patients [10], time to thrombolytics for stroke patients [11], improve cardiac arrest survival [12, 13], enhance response times for emergency cesarean deliveries [14], and enhance staff preparedness for hospital relocation [15]. While these studies compellingly demonstrate the potential of singular projects that use translational simulation, they provide limited insight into how translational simulation programs maintain a sustained presence in organizations. Little is known about how mature translational simulation programs structure their activities, navigate organizational dynamics, and sustain system-level impact over time. Our searches yielded four key studies [16–19] that have investigated translational simulation programs in-depth, offering crucial initial insights on how to launch successful programs. We believe further evidence demonstrating how translational simulation programs plan, adapt, and achieve their intended processes and outcomes will guide simulation leaders to deliberately grow their programs and better substantiate requests for ongoing resources.

Program evaluations involve strategically collecting data to encourage local reflections and practice refinements, while also creating the opportunity to share lessons learned with the broader community [20, 21]. We used a logic model framework (Fig. 1) to frame our interviews of both members of a translational simulation program and the ‘program partners’ they supported within a large, urban academic hospital network in Canada [22]. Rather than using the logic model to generate a prescriptive guide for others to replicate the studied program, we used it as an analytic framework to surface cross-cutting principles that might explain how the studied program functions within a specific organization. Hence, we used the logic model framework to develop an interview guide and sampling strategy aimed at providing simulation educators, leaders, and researchers with transferrable principles that add to and reinforce the available guidance [16–19] for designing, implementing, and evaluating translational simulation programs. We intended for this program evaluation to benefit the simulation community by shifting from conceptual discussions and isolated projects to evidence-informed, actionable models of programmatic translational simulation practice.

**Fig. 1:**
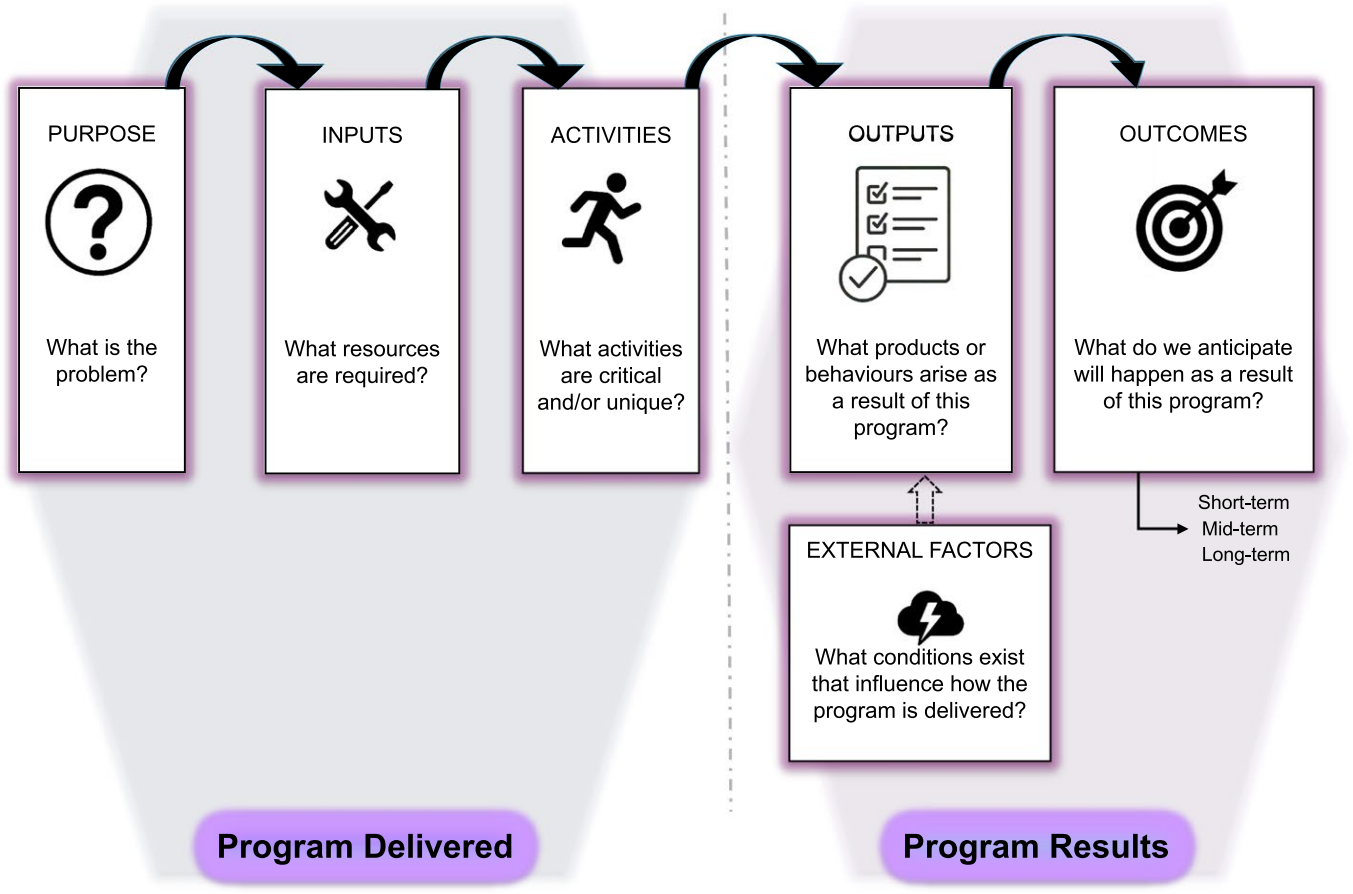
Representation of key elements of the logic model evaluation framework

## Methods

### Study design

A logic model evaluation approach typically involves three steps: specifying program elements (e.g., inputs, activities, outputs, outcomes), collecting data to examine how these elements interact within a program, and refining a program theory describing how the program achieves its intended effects [23, 24]. Notably, we acknowledge that logic models merely represent a snapshot of a program and may not capture all of the dynamic ways that contextual factors can rapidly influence how the elements interact. First, we selected an established set of logic model elements from health professions education to collect this snapshot of our translational simulation program (Fig. 1) [25]. Second, we collected interview data to build an initial set of claims for each element of the logic model [24]. Third, we adopted a team-based approach to propose a refined set of claims to form a ‘program theory’ about how our translational simulation program has realized its goals to implement planned services and activities well (i.e., achieving its theory of change for serving its target population) [26]. 

We recognize that traditional outcomes-focused logic-model evaluations emphasize granular specification of program elements to support replication [27]. In intentionally diverging from that tradition, we position translational simulation programs as operating in resource-dependent contexts, meaning we believe that no other organization can replicate our program’s exact structure, policies, staffing model, or dynamics with partners. Thus, rather than aiming to produce a granular, mechanistic logic model of ‘how’ our program works, we used interview data as the primary source to access how stakeholders uniquely describe ‘how’ and ‘why’ program inputs, activities, and contextual factors interacted over a five year period. Our approach brings value by providing rich, stakeholder-level narratives that simulation leaders can use to reassess existing strategies, reconsider program goals, and refine practices, while fostering shared understanding and clear communication of the program’s purpose and progress [21]. 

We adopted a realist approach to guide this evaluation. A realist stance assumes that program structures and outcomes exist yet can only be imperfectly understood because they are situated in complex social systems [28]. From this perspective, a logic model provided a structured way to define program elements, and the interviews provided data for exploring how program team members and partners worked together via these elements in practice. This realist approach supports researchers to pragmatically use multiple methods to understand not only *what* occurred, but *how* and *why* program processes functioned within a complex healthcare system [28, 29]. Ultimately, we used logic model elements as *sensitizing concepts* for a qualitative inquiry aimed at ‘theory refinement’, rather than at procedural replication.

### Study context

We aimed to identify and explore the linkages between the purpose of Unity Health Toronto’s translational simulation program, its multiple elements, and its sequences of activities and outcomes. Unity Health Toronto provides quaternary care through its network of hospitals including St. Michael’s Hospital, St. Joseph’s Hospital, and Providence Healthcare (representing 1578 beds > 12,500 staff and physicians and > 6300 learners). As part of the already established Unity Health Toronto Simulation Program, the Translational Simulation program began in 2019, with the appointment of a program lead (AP). As part of their full-time roles committed to the entire Simulation Program, staff (four full time simulation educators, 3.5 full time equivalent simulation specialists, one program coordinator, and one manager) support incoming projects. The simulation educators provide project planning, oversight and delivery, in collaboration with key stakeholders, while the simulation program manager provides operational and strategic guidance. Simulation specialists and program coordinators are used ad hoc, as schedules permit.

Over that time, we note that the translational simulation program team has accumulated much experience; for example, the program typically completes three to five projects per year, with 8–12 participants on average, with up to 30–40 participants for large projects. Projects in our context are defined as a time bounded initiative that uses simulation to diagnose or intervene in real-world healthcare systems with the explicit aim of informing practice, policy or design decisions. Each project is unique in its partners (administrative, vs. clinical) scope (one-time intervention vs. a series of scaffolded simulation activities), and purpose (designing and/or testing spaces, policies and processes). We believe that an evaluation would offer valuable insights into how translational simulation programs can be purposefully structured to align with health service priorities.

Ethical issues can arise when evaluators are closely tied to any program, potentially leading to misrepresentation or exclusion of stakeholder voices [30]. Considering this risk, our chosen evaluation team (RB, AT, and NC)—uninvolved in implementing the translational simulation program activities or decision-making— led the study recruitment, data collection, and analysis. We received approval from our organization’s institutional review board responsible for quality improvement projects, Review of Quality Improvement Studies (ReQuIST).

### Participants

We accessed the translational simulation program’s database to identify key organizational user groups (i.e., partners) who had accessed and collaborated with the program from 2022 to 2024. Using email, we recruited participants willing to volunteer in the study from two groups. First, we focused on the translational simulation program’s internal team members, which included three program leaders, four educators, two specialists, and one coordinator. Second, we recruited partners employed at Unity Health Toronto who had engaged with the program to complete a project in the relevant timeframe. Data collection spanned 12-months (December 2023- December 2024) to allow any ongoing projects to reach completion, where possible.

### Data collection: phase 1

**Fig. 2:**
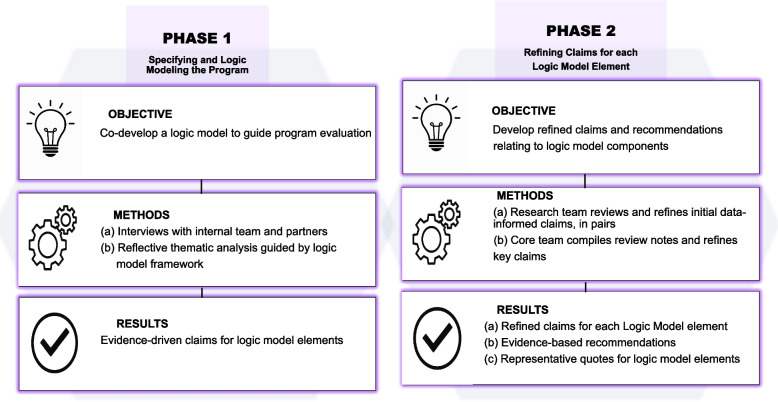
An overview of data collection and analysis methods across study phases 1 and 2

This logic model evaluation unfolded across two linked phases (Fig. 2). In phase 1, two research coordinators used two separate interview guides (Appendices 1–2) to conduct an approximately one-hour, one-on-one interview with members of both groups via video-conferencing software. All interviews were audio recorded, transcribed with participant’s verbal consent, and deidentified for analysis.

Our interviews with internal team members helped gather perspectives on the translational simulation program’s priority matrix for selecting projects, roles and responsibilities, implementation processes, reporting practices, and the perceived impacts of projects. As an integrating data source, our interviews with partners focused on capturing their perspectives on onboarding with the program, building a shared vision for project activities, communications and reporting mechanisms, outputs and outcomes tracking, and future planned engagement with the program*.*

### Data analysis: phase 1

Three core team members (AT, NC, RB) used a reflexive thematic analysis [31] to analyze all 24 participant interviews using a predominantly deductive approach informed by the selected logic model elements. We followed the established six phases of reflexive thematic analysis (i.e., data familiarizing, generating initial codes, generating themes, reviewing themes, defining and naming themes, and reporting) [31, 32], recursively and iteratively, More specifically, in using the chosen logic model elements (Fig. 1) as sensitizing concepts, we focused less on generating and naming themes and more on refining our understanding of the dynamics of participants’ perceptions and reported behaviours relating to each logic model element. That is, we also analyzed the interview data inductively, attending to how the initially ‘static’ logic model elements became ‘dynamic’ in terms of participants’ rationales, mechanisms, and challenges they described encountering and working through to accomplish each project’s goals. Additionally, participants’ responses in early interviews informed later interview questions, and ideas generated during later interviews shaped re-analysis of early interviews, supporting a concurrent, iterative approach to data collection and analysis.

The core team (AT, NC, RB) used NVIVO v12.0 (QRS International Pty Ltd, Doncaster, Victoria, Australia) to organize and code all transcripts from all participants. We met biweekly to discuss the analysis as it progressed from phase to phase. In two final team meetings, the core team reviewed and organized the themes as logic model elements. Our ongoing team-based analysis enhanced credibility, dependability and rigour [33]. 

### Data analysis: phase 2

Following the initial deductive coding and familiarizing and initial coding phases of our analysis, the core team formulated initial key claims to explain how the translational simulation program functioned effectively in relation to each logic model element. For example, when considering program inputs, our initial claim stated that team members had a: “*Shared understanding, role clarity and clear team capacity to select and mobilize resources to implement the plan generated during the intake meeting.*”

After forming these initial claims, the core team invited all research team members to meet in pairs to review whether and how the data coded in the NVivo database for each logic model element (~ 10–20 pages of quotes per element) could be used to refine or refute the initial claim. We purposively paired one core team member with an internal program member who also met authorship requirements (Purposes = NK & AT; Inputs = KS & NC; Activities = CL & AT; Outputs = LB & RB; Outcomes = AP & RB; Unintended Outcomes = AP & NC; External Factors = NK & NC; Miscellaneous = LB & AT). Each pair spent approximately two hours meeting to discuss their independent review of the relevant data, how they interpreted the claim, and came to a consensus on any revision to the initial claim. Each pair kept detailed notes of their process for auditing purposes to enhance trustworthiness [34].

Lastly, the team met twice to review the meeting notes and claims for each logic model element, resulting in three primary outputs (see Results). As a team, we reviewed the refined set of claims, and formulated a set of pragmatic recommendations aimed at supporting other translational simulation program leaders. Specifically, we framed these to help shape transferable *processes* (i.e., adaptive implementations of planned services and activities) and transferable *outcomes* (i.e., an adaptive theory of change to serve their target population effectively) [24, 26].

### Reflexivity statement

Our evaluation team included a team of clinicians, education researchers, and simulation scholars and practitioners. As clinicians, the team represents many professions including physical therapists (LB, CL), nurses (NK), physicians (AP), and respiratory therapists (KS). As scholars, the team has expertise in translational simulation program conceptualization and development (AP, LB, NK), scenario design and implementation (CL, KS, LB, AP), research on simulation in healthcare (RB, AP, LB), and qualitative methods (RB, AT, NC). As simulation program staff, the team included directors and managers (AP, NK, LB, RB), simulation educators (CL, KS), and education research coordinators (AT, NC).

## Results

**Fig. 3:**
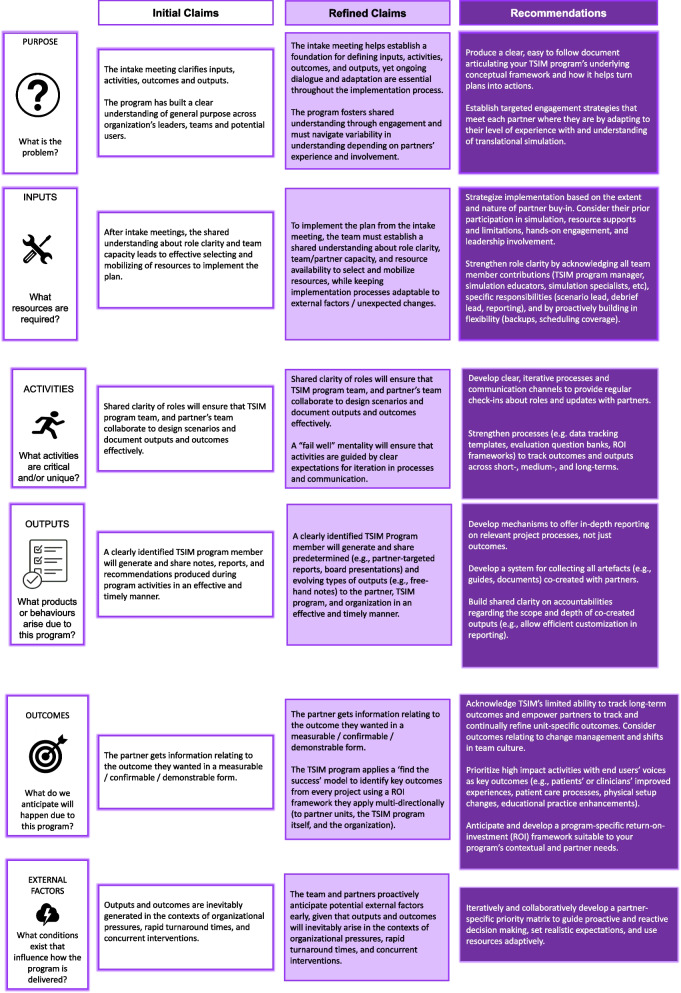
Initial and refined translational simulation program claims relating to each logic model element, with associated recommendation

Our interviews with internal team members (*N* = 10) and program partners (*N* = 14) represented the work of 11 translational simulation projects. Traditionally, many researchers display their logic model findings in tabular form, however, we chose to provide a brief narrative below and to represent our findings through selected participant quotes (Table 1), and a visualization of our initial claims and refined claims as we modeled the data for each logic model element for our translational simulation program (Fig. 3). We also visualized our findings through an analogy to cycling, which we share in Appendix 3 for interested readers.

**Table 1:** Representative quotes from internal team members and program partners, organized according to each logic model element

Logic Model Element	Reference Quote (Internal Team)	Reference Quote (Program Partner)
Purpose	*“We take on majority of the projects…. we've had situations where they [partners] either don't actually understand the problem that they have, [or] don't have the resources to dedicate to work in partnership…we've said no to those because we can't do it alone. And no one's going to win by only half doing it.”* – IM5, on the criteria for collaboration *“I think we all look to each other for different strengths that we have. So, I think that works really well for us. If… there was something that we just were really stuck on, we would bring in [the program leader]”* – IM9, on developing shared understanding	*“We had a bit of a sense of what we wanted to achieve with [the program]… what it can provide became more clear when we first connected with the [program] team. And so to see the scope of what services can be provided… options we had in terms of both the implementation and… the consolidation of feedback and the evaluation process…those are some of the details that we were connecting with [the program] directly”* – P9 Operation Readiness Specialist, on their purpose for collaboration and contents of the intake meeting *“…there is a ministry of labor requirement that we put in screening and alerting behaviors. But then we have this opposite kind of requirement in practice, to not just keep our staff safe, but keep our patients safe from harm. And being labeled as violent…, can have significant harms, right. And so… [the program helped in seeing] if we can bridge these two different kinds of… perspectives into something that is workable… I’m gonna say like an olive branch on how to move forward.”* – P4 Senior Director, on their clear goals of leveraging the program as a neutral body aligning perspectives
Inputs	*“Translational simulation program… team, by and large, are motivated people who are passionate about delivery of care and see the value in… process-oriented outcomes… that's the number one strength. I think, the strength is that we have a large simulation program that's funded by the hospital, not many places have that.”* – IM3, on program strengths and seeing the program as better positioned with resources and institutional backing *“The simulation educators attend, sometimes… the Medical Director attends [intake meeting], it is very much project specific. And we'll go through… its way more detailed… who do you think should be involved?… What resources do you think you need?… go through that whole spiel… we always send summary emails… lay out next steps like, Okay, so the SIM team is going to do A, B, and C, our partners are going to do one, two, and three at this time, we're going to come back together, and… move forward from there… it takes a lot of really strong communication.”* – IM5, on establishing shared understanding and decision-making	*“One of the standards in IT security is to routinely do tabletop exercises… and then test how our organization will respond accordingly. And so, it was through our leadership team, executive leadership as well, that suggested… expertise within the hospital that does this on a regular basis. And so, there’s an opportunity to leverage that expertise… And also, we did get guidance from our… board of directors.”* - P3 Project Manager, on their goals of leveraging institutionally accessible expertise for routine tabletop exercises *“So our focus was more on the system impact and understanding like the human movement and logistics… as opposed to like high fidelity of what their vital signs are doing…, it wasn’t about the medical management, like the way that other sims run. So that was something that I remember took a little while to explain and understand.”* – P7 Manager & Quality Assurance Specialist, on challenges in establishing shared understanding
Activities	*“I prefer sometimes to just hop on a call, rather than solve things over email. And I guess it also depends on how complex it is, and how many people are involved… and it can be really hard… to book a meeting with everyone at the same time. So, in that case, then in emails might be the best… we'll try to minimize the amount of communication, if it involves a lot of groups, because then… too many cooks in the kitchen, if you will.”* – IM9, on pragmatic communication strategies *“There's this program package that… It doesn't work well for me… In my head, because things live in different areas that's an opportunity for errors… because we're so flexible, sometimes I can see how things can get missed because we all work on projects… with a different project monitor. But whoever's the primary on that project, somehow is able to… keep going with it. So, we don't use Gantt charts because the deliverable and actionable items are nuanced… But we do use a template to help us plan for the output report… But when it comes to all the other formatting, I find it hard.”* – IM7, on challenges and strategies in project monitoring and reporting	*“I think everyone wanted to boil the ocean and do everything. And they really helped us narrow the scope into something that is very realistic… perhaps more realistic. So, they helped define the objectives… pulled in hospital expertise… we relied entirely on their expertise to write the scenarios… then the rest of us reviewed it. So that was like, they own that piece as well, which is great.”* - P3 Project Manager, on roles and responsibilities collaborating with the program *“Most of our communication was *via* email. We did have a couple of brief zoom meetings; we ended up filling out an intake form or some type of form afterwards that kind of summarized our thoughts about it… No issues with the communication, it was really easy to reach both of the team members… the follow up sheet or the intake form was pretty thorough… they did a really good job… organizing a large group of individuals, like we had the entire MS ICU, the OB staff that comes down for codes as well. So yeah, they did a did a good job.”* - P10 Clinical Operations Lead, on Program’s communication and coordination with different stakeholders
Outputs	*I create annual reports for the entire program, that translational simulation is a part of, but it's not all of what we do… we've used it for foundation pitches before to get extra funds. And… it's so important to have rigorous documentation… we even save email trails from our users, particularly the ones where they're like, this is amazing, we couldn't have done this without you… you can see the iteration from the annual reports at a high level. And then if we need something specific, we can go in and pull out that one piece and send it to them.”* – IM5, on Program documentation and reporting *“We feel like we've met our objectives, like, based on how the simulation ran, like, were people immersed?… Did they feel engaged? Were they participating? Did they seem excited? Because often the translational simulations that we run… we want to engage the point of care staff. We're changing things in the organization that we want their opinions on. And so did we get that engagement I think is also really important.”* – IM9, on determining project’s success	*“We need to better document like actual metrics or key performance indicators. And whether that boils down to even as simplistic as pre session, how comfortable would you be if this happened in terms of you knowing what to do on a scale of one to five, and then post session to prove the value of the simulation to the audience, as opposed to just having a final report?”* – P2 Manager, on suggestions to better Program reporting *“So, their report, we read it. And we said, this is… way too long, it should be way more concise. It should have some key takeaways. It was pages and pages long, and it was nothing that we had not heard before.”* – P5 Project Manager, on Program’s reporting
Outcomes	*“Yeah, I think I think [the program is] a jewel of our simulation program. And so, I think what we have to do is just make sure we continue to prioritize high impact activities with patient voice as the outcome. And I think if we continue to do that, we’ll be successful”* – IM3, on Program’s reputation and reinforcing principles *“The strength is that it's being utilized frequently by users… the proof is in the pudding… people are using it and they're requesting it, they are interested in learning more.”* - IM4, on the growing Program uptake *“But I do think that there's an opportunity for us to connect with other hospitals who maybe don't have access to simulation… we have the ability to help other organizations, knowing that we might be the only people that are doing this. So, I would like to make more of an impact not just at our organization, but at others… also recognize that our team is small and… we just don't have that capacity right now.”* – IM2, on Program’s vision and broader impact	*“There’s definitely a return on investment. Because of that simulation, the team who we were borrowing the space from requested their own simulation… so I was just like… this is who you kind of connect with to get that ball rolling. And then, our staff were involved in their simulation as well, because we’re literally in the same space.”* – P10 Clinical Operations Lead, on the return of investment of collaborating with the Program *“I think there were huge impacts… I can look back to numerous examples where we made significant changes in the design and alterations, prior to opening that we wouldn’t have been able to do without getting this feedback… and seeing it play out in, in a simulation format. So, there are substantial… changes that we’ve made based on feedback we’ve received from the [program]”* – P9 Operation Readiness Specialist, on Program’s measured impact

### Purpose

Our analysis showed that the purpose of collaboration between the translational simulation program and its partners typically emerged during initial intake meetings, where both sides described working to build a shared understanding of the partner’s needs and the translational simulation program’s capabilities and limitations. These meetings often concluded with an initial simulation plan with clearly outlined roles, responsibilities, and reporting structures. Both internal members and partners reported that disputes and communication disruptions could arise at this stage, requiring investments of effort and time to build familiarity, trust, and an open dialogue. In most cases, disputes were resolved, though some diffused beyond this stage, leading to more meetings to develop a shared vision, an ongoing reassessment of needs, or a mutual decision to discontinue collaboration.

### Inputs

While many partners expected that the translational simulation program team would spearhead most project stages, more active collaboration tended to emerge over time as processes became clearer and the program team’s and partners’ capabilities became evident. Resources were selected and mobilized depending on every partner's level of commitment and resources, and how the project aligned to the program's priority matrix. We found that the translational simulation program team reported regularly managing tensions between internal workflows and pressures and ensuring the partner’s buy-in was maintained. Both internal team members and partners described the challenges of establishing shared understandings of role clarity and team capacity when it came to finalizing decisions about resource allocation. Once resolved, the translational simulation program team’s shared understanding with partners appeared to guide key decisions about who: developed simulation scenarios, assumed which roles (planning, participating in the simulation etc.), managed physical spaces, and governed ongoing allocation of resources. Notably, a few partners mentioned that despite investments in developing shared purpose and in resource allocation decisions, disagreements could continue to diffuse into the remaining logic model elements due to external factors (noted below) or unexpected deviation from shared understandings.

### Activities

All participants suggested that the process of shifting plans into actions required commitment to shared decisions, and to open dialogue and adaptability. The translational simulation program team reported needing to communicate clearly about assigned roles, engage with project management systems, and adapt reporting practices to meet evolving needs (e.g., shifting scenario designs in real-time, customizing reports to better align with partner needs). Participants tended to agree that clear, timely communication about roadblocks helped to uphold their shared vision, though some mismatched expectations were inevitable. For instance, some members of the translational simulation program team reported instances where scenario updates would not reach all team members, leading to some feeling uninformed and upset during implementation. Though these lapses rarely stalled progress, they did cause occasional uncertainty.

### Outputs

We found that the translational simulation program has regularly been responsible for providing outputs to partners and for its own records, including simulation scenario debriefs, tailored reports, and need-based follow-up documentation. From the partners’ perspective, outputs generally aligned with their expectations and impressions formed during the activity, and often exceeded them. However, some partners reported wanting more than descriptive summaries of completed activities, including deeper analyses and decision-making recommendations. Translational simulation program members noted that they may not be positioned to conduct such analyses nor offer such recommendations, given they lack insight into each unit’s unique pressures, accountabilities, and budgets. Team members and partners both appeared to sense that outputs have a variable depth of possibility and sensed a need to reflect on achieving ‘the right depth’ in future projects.

### Outcomes

As a principle, the translational simulation program has aimed to achieve measurable outcomes in all projects. Participants reported a variety of short-term outcomes, including cost savings (e.g., altered equipment purchase plans), improved self-reported team wellness, and refinements to policies and rollout plans. Reported mid-term outcomes included identifying latent safety threats to inform architectural planning, sequenced changes to care processes and/or educational practices, and continued refinement of the program’s own operations. While long-term outcomes can be difficult to attribute directly to brief translational simulation interventions, positive evaluation scales, repeat users, and a strengthened reputation appeared to reflect the program’s lasting impact.

### External factors

Participants framed the translational simulation program as operating within complex, high-pressure environments shaped by tight deadlines, overlapping priorities, and site-specific demands. Organizational pressures (e.g., policy rollouts, construction timelines, and regulatory requirements) often pushed the translational simulation program team to respond quickly, limiting their ability to plan proactively. Concurrent interventions and competing partner demands often shifted focus, which could prevent deeper exploration of critical issues. Additionally, the translational simulation program often had to tailor simulations across diverse clinical sites due to varying priorities, which further complicated implementation. Indeed, many of our findings indicated that the program team needed to regularly adjust course in real time, often under resource constraints, to stay on track.

### Analytic synthesis: foundational and identified principles

As we analyzed data for each logic model element, some principles about how this translational simulation program tends to operate appeared to permeate our findings. As a set of a priori intentions, we found that the program was originally designed and ultimately received by partners with a ‘make it a success’ entrepreneurial perspective that helped it function like a startup company. That is, all participants tended to report an approach where the team did not wait for perfection, choosing to pilot ideas rapidly, take safe risks, and dissect failures to encourage ongoing, agile team-based improvements. The translational simulation program’s use of a ‘design thinking’ conceptual framework [35] likely contributed greatly to these perceptions.

Other identified principles we observed included participants repeatedly noting that the translational simulation program represents a position of neutrality within the organization; for example, in some cases, the program’s processes, outputs and outcomes helped partners make decisions that had been previously stalled by political stalemates. An additional observed property of the program was that the team’s regularly reflexive activities (e.g., summarizing outcomes in annual reports) to capture each project’s key learnings and outcomes has led it to develop a robust ‘return on investment’ (ROI) framework. The program team now uses that ROI framework to guide intake meetings, shape output and outcome priorities with partners, and position the value of the program at Board meetings and other organizational functions.

## Discussion

Our logic model program evaluation unveiled numerous insights about how the Unity Health Toronto translational simulation program has succeeded and about where it can invest effort and reflection toward continuous quality improvement. Collectively, our findings suggest that translational simulation programs can function as an organizational capability that enables healthcare system leaders to diagnose and address system-level challenges.

Analogous to how simulation facilitators debrief participants after sessions to promote learning outcomes, our evaluation reinforces this best practice of structured reflection. Our findings build on foundational reports on how to setup a translational simulation program successfully [17, 18], adding significant detail on how to sustain a program across multiple projects and multiple years. We distilled our data-driven appraisal of our logic model evaluation elements into a refined list of claims and related recommendations (Fig. 3). In addition to a blueprint offering transferable principles and pragmatic activities that other translational simulation programs might consider adopting, we interpret our key findings below. We found that program was originally designed to align intentionally with frameworks describing how practitioners can move from concepts to action in translational simulation [3, 4, 35]. The program has framed translational simulation as highly context-sensitive, whereby a principles-based rather than prescriptive model optimizes workflows. This approach enables simulation activities to be tailored to the unique needs, constraints, and goals of each program partner’s setting, thereby enhancing relevance and impact.

Our observed high variability in project inputs seems to underscore the need for translational simulation programs to exhibit flexibility and agility, especially during the early phases of partner engagement. Furthermore, our findings highlighted that differences in expectations manifest regularly, suggesting the need to prioritize communicating with partners early and often.

Another recurring finding was partner requests for additional outputs, deeper analysis of project outcomes, and continued evaluative support. Such requests may lie outside of the translational simulation program team’s expertise and responding to them would come with an opportunity cost to other projects. Navigating this tension requires that translational simulation program leaders tread carefully to ensure that specific partner expectations are met without compromising overall program efficiency. For example, our proposed refinement to how to establish “purpose”, from an initial intake meeting to an ongoing process of dialogue and adaptation, echoes the proposal that translational simulation adds value most when embedded in iterative cycles of creating a sense of urgency, planning, implementing and improving [17, 36]. This tension in expectations regarding outputs reverberates as the translational simulation program seeks to be viewed as a value-add within the broader organization. The translational simulation program team’s decisions not to take on tasks beyond the core scope must be carefully balanced against maintaining a strong reputation as a strategic manager of resources that support high-value partnerships.

Like other studies, the evaluated translational simulation program appears to have demonstrated success in shaping healthcare teams’ performance, clinical outcomes, and system-level change [22, 37, 38]. Despite such successes, however, participants’ regularly cautioned that failing to integrate structured follow-up and project-specific metrics could result in translational simulation programs overpromising potential outputs and outcomes. Such instances could then jeopardize future initiatives and opportunities through negative impacts on the translational simulation program’s reputation, as well as the internal team’s and the organization’s trust in the process. To mitigate these risks, we put forth recommendations to build shared clarity on expectations regarding the scope of outputs and the customization of reports. In addition, we recommend that translational simulation programs implement processes that clearly define, justify, and track mid- to long-term outcomes, which we believe will yield dividends at both the simulation program and organizational levels. Indeed, our program and others like it may benefit from seeking increased balance between investing more resources in longer-term monitoring and implementation support for fewer projects, versus initiating numerous projects without sustained follow-up. We are not aware of research offering guidance on who should be responsible for producing and measuring outputs and outcomes in translational simulation projects, and our findings suggest that these tensions deserve further investigation. Until stronger evidence arrives to guide the process, translational simulation programs should prioritize early discussions with stakeholders to clarify project outcomes, outputs, and the responsibilities associated with each.

Like prior studies of other simulation programs in Australia and North America [19], we found that the evaluated program exhibited a distinctive blend of properties that are typically ascribed to startup companies (and some not): growth oriented, innovation-focused, resource-constrained, creative in problem-solving, open to rapidly pilot testing ideas, politically neutral, process-centric and a tendency to over-commit. We are not the first to report such characteristics, which leaders of other simulation programs have also noted [19]. Our replication and extension of that work may support the simulation community in developing guiding principles for launching and sustaining translational simulation programs. For example, researchers could further explore how simulation programs can maintain neutrality during organizational political stalemates, given the likely scenario where some partners could feel ‘threatened’ that the program may provide insights that conflict with their priorities. As another example, program leaders could explore how startups in other industries have achieved success and managed challenges given the analogous approaches to problem-solving and rapid growth [19]. Learnings from our data may also include how to an evaluation can inform translational simulation program leaders on how best their unique program can demonstrate ROI, cost-effectiveness and other tangible outputs to key partners and organizational leaders. Interested readers may refer to early papers [39, 40], and more recent ‘value-based simulation’ models that advise aligning what programs measure with what stakeholders value and find meaningful [41]. Much like startups must demonstrate their value, translational simulation programs must regularly reflect on how to demonstrate their value and impact within their organization and funders. Beyond building a vision for the translational simulation program, this “startup” mentality may also inform hiring practices, favoring those who can work within this agile and innovative environment. [19]

Our project had limitations and strengths. For example, our use of outsiders to collect data and conduct initial analyses aligns with views that evaluators benefit when partnering with staff to consider programs from multiple perspectives [24, 42].Alternatively, use of insiders throughout all research stages may have shifted our methodological and interpretive decisions. We sought to balance our access to both perspectives in our chosen approach of a core team that eventually teamed up with many insider simulation team members to finalize the analysis and data interpretation. We acknowledge that logic models have known shortcomings [43], however, they are also relatively straightforward and we believe simulation program leaders may benefit from applying a similar process to their planning, delivery, and longer-term evaluation phases.

## Conclusions

Fueled by foundational documents articulating concepts [2, 4], and ways to turn concepts into actions [3, 6], we offer data-driven recommendations for how to implement and sustain translational simulation programs. Our interview-based and logic model-informed approach allowed us to surface nuanced mechanisms and tensions, articulate refined claims for each program element, and generate transferrable recommendations to guide practitioners across the healthcare simulation community. We encourage other established translational simulation programs to share their own lessons learned [16–18], and nascent programs to share their aggregated growing pains and successes in real-time. We acknowledge recent successful efforts to affirm how the healthcare simulation community conceptualizes translational simulation (and to avoid the confusing term, ‘transformative simulation’) [4]. With that now established, we believe that the community must develop a collective repository of data-driven insights about how translational simulation programs can continually refine processes toward achieving all facets of ROI that drive innovation and safety in healthcare systems.

## Supplementary Information

Supplementary Material 1.

## Data Availability

The datasets generated and analyzed during the current study are available from the corresponding author on reasonable request.
