# Supplementary material for: Moving beyond projects: a logic model evaluation of an established translational simulation program
Source: Adv Simul (Lond). 2026 Apr 2;11:39. doi: 10.1186/s41077-026-00434-x (PMC13170001; doi:10.1186/s41077-026-00434-x)
Supplement: Supplementary file 1 — Supplementary Material 1. [file 41077_2026_434_MOESM1_ESM.docx]

**Appendix 1: Interview Guide for Internal Members**

Do you consent to record and transcribe this Zoom meeting? Do you consent to participate in this debrief?

1. Tell us your current relationship with the Translational SIM Program.
2. How are you currently related to the Program? What are your responsibilities in your current role?
3. How long have you been associated with the Program? Why did you want to be a part of the Program?
4. Can you quantify the duration that you spend working on the program, how much would you say on average do you work on the Program in say like a month?

2. Tell me about your experience in initiating work on partner projects with the Program.

1. In your current role, when do your responsibilities begin when a new project/ partner is considered for the Program?
2. What tasks, resources and team-support do you need as you get actively involved in the projects? Do you always feel supported in when initiating your responsibilities for new projects? Please elaborate.
3. With the Translational Simulation Program team, how has your experience been with communications on briefs and expectations regarding projects?
   - - - 1. *How would you compare the time that you spend on email communications before finalizing roles and responsibilities for a project under translational SIM versus other programs that you are involved with? Do you think this is appropriate, more, or less than ideal?*
         2. *How would you compare the time that you spend on uptake meetings before finalizing roles and responsibilities for a project under translational SIM versus other programs that you are involved with? Do you think this is appropriate, more, or less than ideal?*
         3. *What other activities have been critical or unique in getting your projects setup?(additional staff, negotiations, equipment related conversations, etc.)*

3. Project partners often think they know what they want in their projects. And the Translational SIM program team also surely has its own philosophy about how to do projects well.

1. Tell me about how easy or difficult it is to develop a shared vision with partners on project pathways and project goals?
2. Tell me about how easy or difficult it is to develop a shared vision within the Program team on project pathways and project goals?
   - - - 1. *Which factors most influence the goals and purpose of any given project?*
         2. *How is diversity in opinions handled when you are working through project-related decisions?*
3. We’ve talked a lot about HOW you make decisions about project goals so far. Could you also please talk about WHY you make the decisions you do?

*What are the underlying “ways of thinking” that drives the Program to operate the way that it does?*

4. Tell me about your experience with the Translational Simulation Program with regard to the implementation of different projects.

1. Do you feel the Program has mostly been successful to implement what has been planned for most partner projects? Why or Why not?
2. Have there had to be regular adaptations to the plans, perhaps in the proposed goals, agreed-upon resources, and/or budget? Why or Why not?
3. What kind of project monitoring and reporting system has been in place to keep tabs on the various projects? How does it work?
   - - - 1. *What would you continue doing, stop doing, and start doing differently?*
4. Please tell me about any situations in which there have been arguments, re-negotiations, or cancellations in initially agreed implementation practices, project budget, individual responsibilities, or progress reporting practices?
5. Do you feel that everyone in the Program team works efficiently and fulfils their respective responsibilities as per the plan? Why and Why not?
6. Does the Program face any roadblocks in implementing various projects? What do these roadblocks look like and when do they appear in the project timeline?

5. What kind of documentation does the program engage in, from start to finish during a project timeline?

1. What is your role in keeping timely documentation about projects?
2. What do you feel is currently working with the documentation process? What might you change or refine?
3. Do you know how documents are stored, and to what purpose they are put in the future?

6. Tell me about your experience with the Translational Simulation Program with regard to the outcomes and impact of various projects.

1. How does the team judge whether it has been successful in achieving project goals?
2. Do you feel the Program has mostly been meeting the expectations of project outcomes? Why and Why not?
3. Has the Program ever not been able to meet the outcome expectations for various projects? How so? What do you think have been the reasons for this?
4. How would you describe the impacts of the Translational Simulation Program? What have been the key projects? What has been fundamental in creating this impact?
5. How would you enhance or change the way the program creates impact?

7. PERSONAL Tell me about the reasons for partnering or not partnering with the Translational Simulation Program in future.

1. Do you think your association with the Translational SIM program has been a success? If yes, why? If no, what will make your experience with the Program improve?
2. Has working with the Program made any significant impact on your professional life? How so?
3. Does the commitment to the Program affect your other work commitments in any way? How so?
4. Do you face any hurdles in performing your duties for the Program? If yes, what are these hurdles? In your opinion, how can these be managed?
5. In your opinion, what are the strengths and weaknesses of the Translational SIM program? How can the Program navigate the weaknesses?

8. I have no other questions. Do you have any other comments on the Translational SIM Program?

**Appendix 2: Interview Guide for Program Partners**

1. Tell us about how you became aware of the Translational Simulation Program.
2. What were the channels that helped you in becoming aware of the Program?
3. From your channels/ independent research, were you able to obtain all the information that you wanted on how the Program works?
4. Was there anything you weren’t sure of, which limited your interest in the Program?

2.. How did you think the Translational Simulation was going to help you meet your project goals?

1. Please describe how you initiated your project/s with the Program.
   - - - 1. *What role did you play in initiating and conducting the project?*
         2. *Were other members of your team involved?*
         3. *What resources did you need to leverage from your unit and team?*
2. how did the Translational Sim team members set project expectations?
3. If not for the Program, what were the other options for proceeding with your project?

3. Tell me about your experience in initiating project workings with the Translational Simulation Program.

1. With the Translational Simulation Program team, how was your experience in communicating the project brief and expectations?
   - - - 1. *How many hours did you spend on email for the TSIM project vs. other similar projects were TSIM wasn’t used? Do you think this is appropriate, more, or less than ideal?*
         2. *How many hours did you spend on meetings for the TSIM project vs. other similar projects were TSIM wasn’t used? Do you think this is appropriate, more, or less than ideal?*
         3. *What activities were critical or unique in getting your project setup?*
2. How close did the intake meeting(s) meet your expectations?
   - - - 1. *If you have to rate from 1-5, with 1- being very difficult and 5- being very easy, how was the experience negotiating services and fees with the Translational Simulation Program? Can you please elaborate on the reasons for your rating?*
         2. *If you have to rate from 1-5, with 1- being very difficult and 5- being very easy, how was the experience negotiating reporting conditions with the Translational Simulation Program? Can you please elaborate on the reasons for your rating?*
         3. *If applicable: If you have to rate from 1-5, with 1- being very difficult and 5- being very easy, how was the experience negotiating authorship, research, and individual responsibilities with the Translational Simulation Program? Can you please elaborate on the reasons for your rating?*

4. The Translational SIM program team has their own approach to conducting projects. You and your team also surely had your own philosophy about how to do the project well.

1. What were your initial objectives for the project on your unit? What did you intend to achieve for your project/s?
2. Tell me about how easy or difficult it was to develop a shared vision with the Program on project pathways and project goals?
   - - - 1. *Which factors most influenced the goals and purpose of this project?*
         2. *How was diversity in opinions handled when you worked through project-related decisions?*

5. Tell me about your experience with the Translational Simulation Program regarding the implementation of your project.

1. Do you feel the Program was able to implement the protocol as decided in the intake meeting? Please elaborate.
2. Do you feel the Program was able to implement the protocol with the agreed-upon resources and budget? Please elaborate.
3. Do you feel the Program was able to implement an efficient project monitoring and reporting system for your project? If yes, what did the system look like for your project? Please elaborate.
4. How often did you feel compelled to gather the Program team to revisit implementation practices, project budget, individual responsibilities, and progress reporting practices?
5. Did you face any roadblocks in implementing your program, from start to finish? If yes, please elaborate.
6. Did you engage in, or expect to receive, any documentation about the project from start to finish? How did that process work for you?

6. Tell me about your experience with the Translational Simulation Program regarding the outcomes and impact of your project.

1. How did the Program help you achieve your project goals? Were all goals met? If not, why not?
2. How did the Program and your project team work together? Did everyone meet their respective responsibilities? Please elaborate.
3. How would you describe the impacts of the Translational Simulation Program? What was fundamental in creating this impact?
4. How would you comment on the ‘return on investment’ your unit and team experienced by working with the Program?
5. Beyond any initial outcomes, are there any sustained changes on your unit resulting from this project? If so, please tell us about them? If not, why not?
6. How would you enhance or change the way the program creates impact?

7. Tell me about the reasons for partnering or not partnering with the Translational Simulation Program in future.

1. Do you think your partnership with the Translational SIM program was a success? If yes, why? If no, what in your opinion would have made the partnership successful?
2. Based on your experience how would you advise the program on:
   - - - 1. *What to continue doing?*
         2. *What to stop doing?*
         3. *What to start doing differently?*
3. Would you consider partnering with the Translational SIM program again in the future? If yes, why? What kind of project would you imagine contacting them about?
4. If no, what should the Translational SIM program work on so that it offers you more appropriate services for your project needs?

8. I have no other questions. Do you have any other comments on the Translational SIM Program?

**Appendix 3**: Depiction of our study findings analogized as a peloton of cyclists navigating challenging paths to reach a desired outcome


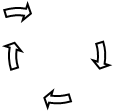

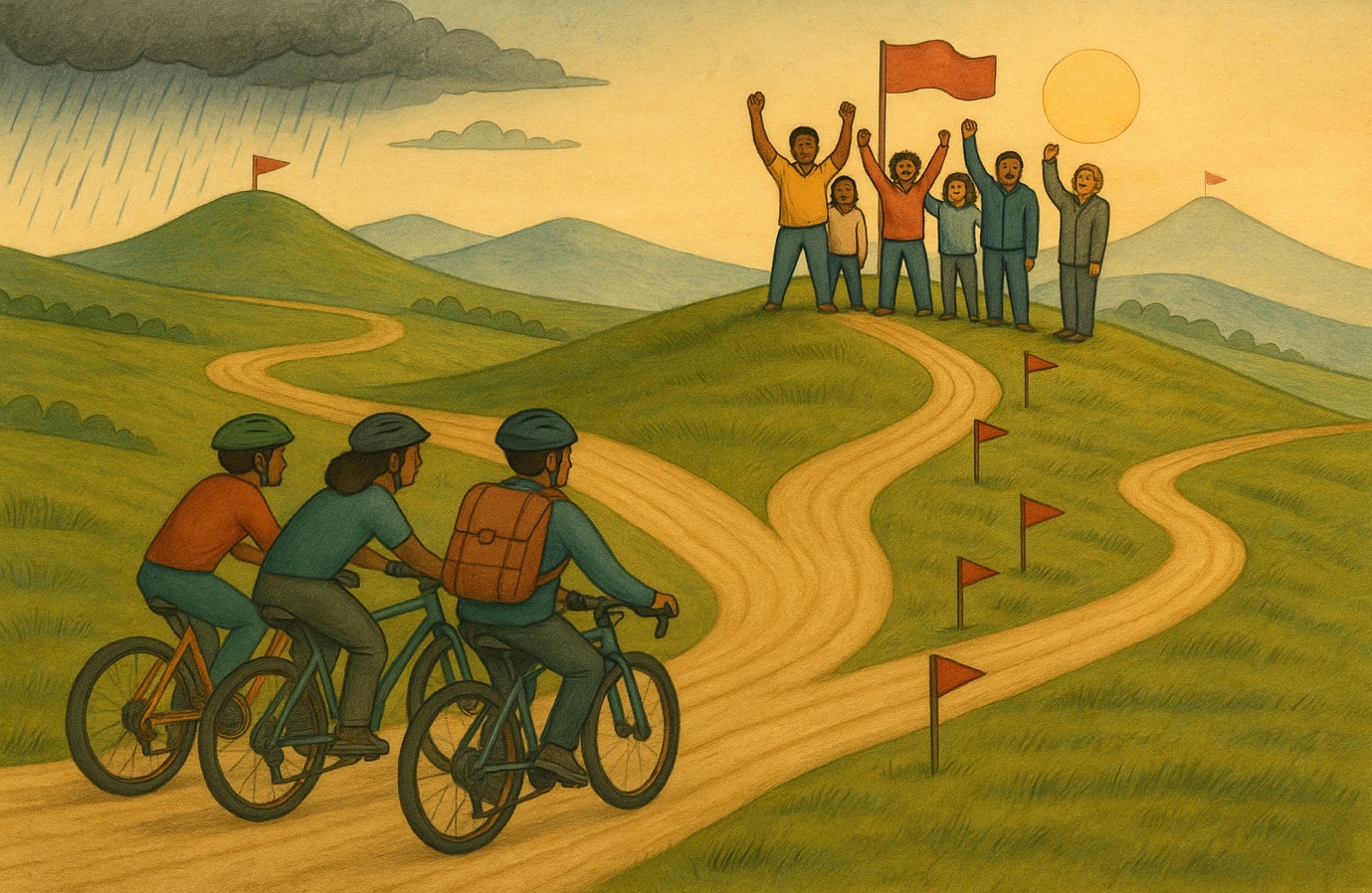


**Activities**

**External factors**

**External factors**

**Outputs & Outcomes**

**Purpose**

**Inputs**

The scene depicted in Appendix 3 aims to represent, imperfectly, the complicated and integrated logic model findings about the translational simulation program: the lead cyclist wearing a backpack (program team) guides its team, the peloton of cyclists (program partners) to set out toward a hilltop, having developed a shared sense of the destination and its significance (Purpose). They will also rotate the lead role as they proceed, demonstrating distributed leadership. To reach the hilltop, the peloton must set and navigate toward checkpoints (Activities) and work through expected and unforeseen challenges (e.g., missed checkpoints or wrong turns), symbolized by rough terrain and shifting weather (External factors). The cyclists use resources they have planned to have on hand to adapt along the path (Inputs), including their own pedaling power (e.g., working harder to push over hills, shifting pace to navigate bumps in the road).Their journey culminates in reaching the hilltop, where the cyclists can evaluate the balance of how their effort investments aligned with their perceptions or immediate measures of how the journey went (Outputs), and review measures of their success (Outcomes), providing insights for their future journeys, motivation for others who may want to join the peloton (e.g., other program partners), and a plan for accomplishing future longer term goals (i.e., the hilltop and flag in the distance). The spectators represent other members of the organization who learn more about the translational simulation program through its successes with each program partner.
